# Reference intervals for peripheral blood neutrophil CD64 index and monocyte HLA-DR in healthy adults

**DOI:** 10.1038/s41598-026-42826-z

**Published:** 2026-03-05

**Authors:** Liangjun Zhang, Yi Li, Huixiu Zhong, Jingyuan Huang, Minggang Yin

**Affiliations:** 1 Department of Laboratory Medicine, Zigong First People’s Hospital, Sichuan province, Zigong, China; 2 Department of Laboratory Medicine, West China Hospital, Sichuan University, Chengdu, China; 3 Department of Laboratory Medicine, Zigong Fourth People’s Hospital, Sichuan province, Zigong, China

**Keywords:** Neutrophil CD64 index, Monocyte HLA-DR, Reference intervals, Healthy adults, Infectious disease, Immunology, Infection

## Abstract

Neutrophil CD64 (nCD64) index and Monocyte HLA-DR (mHLADR) played an important role in the diagnosis of infection, however, reference intervals for these parameters in healthy adults remain insufficiently defined. This study aimed to established reference intervals for nCD64 index and mHLADR% in the peripheral blood from 285 healthy adults. All the subjects were grouped into subgroups according to sex (male and female) and age (20–51 years, 51–90 years). The analyses indicated age to be an important factor associated with changes in nCD64 index, which gradually increased from 20 to 90 years and showed a positive correlation with age (P < 0.0001). In contrast, no significant differences in mHLA-DR% were observed across gender or age groups (p > 0.05). Furthermore, no correlation was found between nCD64 index or mHLA-DR% and all the inflammatory derived indicators of blood routine (P > 0.05). In conclusion, the reference intervals established for the nCD64 index and mHLA-DR% in healthy adults may provide supportive information for monitoring immune status in the context of infectious diseases.

## Introduction

Neutrophil CD64 (nCD64), also known as the high-affinity Fc-gamma receptor I (FC-γR1), is a transmembrane glycoprotein belonging to the immunoglobulin superfamily. It is constitutively expressed on monocytes, macrophages, and dendritic cells^1^, while its basal expression on resting neutrophils is low. Upon inflammatory stimulation, nCD64 is rapidly upregulated and participates in immune regulation^2^, it can be used as a valuable marker for the diagnosis of infection, disease monitoring and prognosis evaluation of infectious diseases^3^. Human leukocyte antigen-antigen D related (HLA-DR) is a protein required by antigen-presenting cells to present antigens to T cells, and is expressed on monocytes, macrophages, and B lymphocytes. Increased HLA-DR expression reflects the activation of immune cells, while decreased HLA-DR expression indicates impaired antigen presenting capacity^4^. Monocyte HLA-DR (mHLA-DR) is critical for the initiation of adaptive immunity, and its low level is regarded as a marker of immunosuppression^5^. During infection, reduced mHLA-DR expression inhibits antigen presentation and immune response, leading to immune dysfunction. Its detection is helpful to assess disease severity and prognosis^6^, and monitoring mHLA-DR level also plays a good guiding role in immunoregulatory therapy.

The diagnostic and predictive value of elevated nCD64 and reduced mHLA-DR in infectious diseases is well established. However, standardized reference intervals for these biomarkers - particularly those accounting for potential variations across different age groups - are currently lacking. The absence of well-defined, age-specific reference intervals complicates the clinical interpretation of results, which may lead to either over-diagnosis or under-recognition of infection. Therefore, the present study aimed to establish reliable, age-stratified reference intervals for the peripheral blood nCD64 index and mHLA-DR% in a well-defined cohort of healthy adults. These intervals are essential to support accurate clinical decision-making and improve patient management in the context of infectious diseases.

## Material and methods

### Patients

**Fig. 1:**
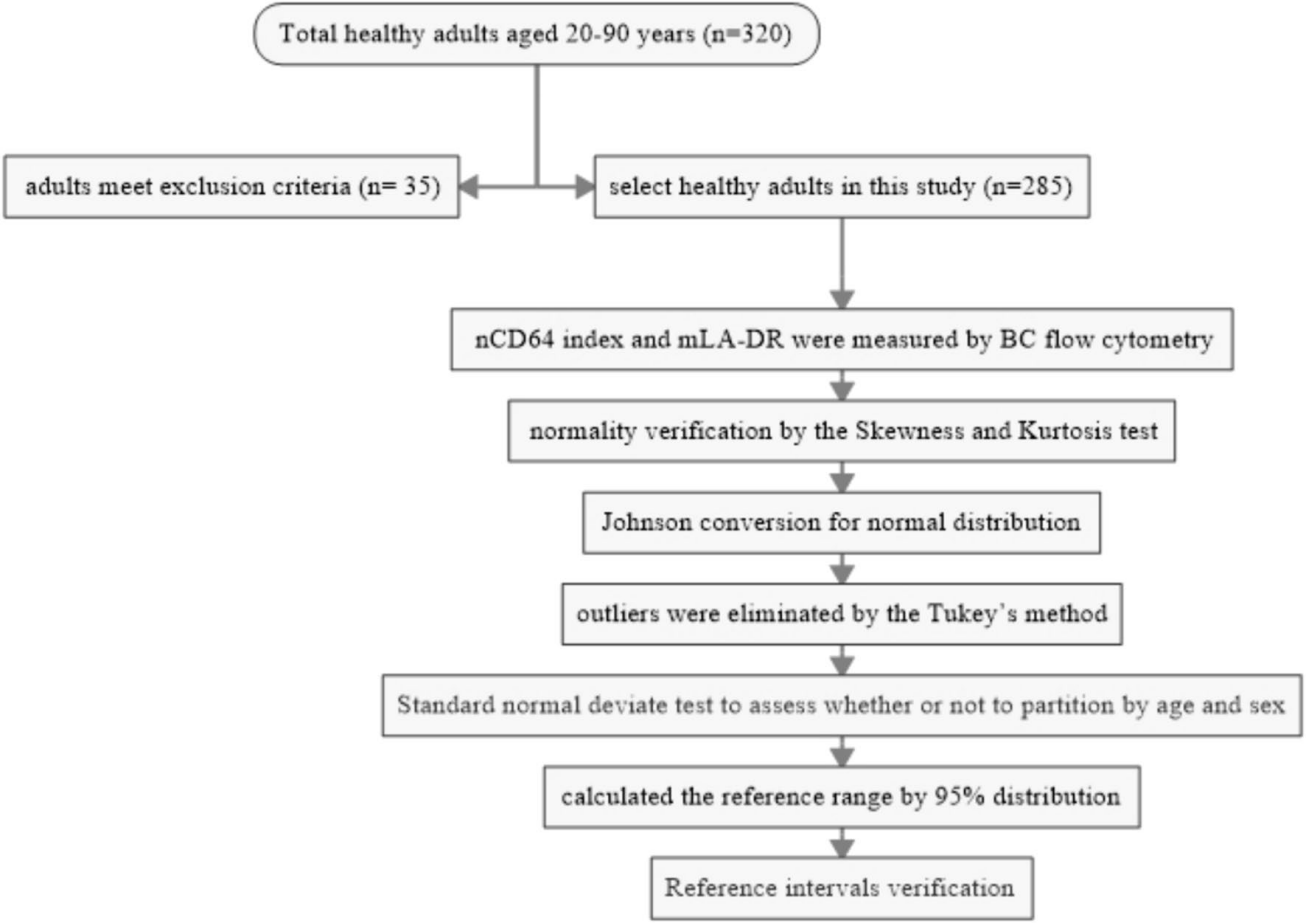
The flow chart of this study for establishing reference intervals.

Participant recruitment for this study was conducted from February 1, 2024, to February 1, 2025. A total of 320 healthy adults aged 20–90 years were initially recruited during physical examinations at Zigong First People’s Hospital. The following exclusion criteria were applied to define a healthy reference population: Abnormalities in routine blood tests (e.g., leukocytosis, leukopenia, anemia, thrombocytopenia). Abnormal liver or kidney function tests, specifically: Liver function: Alanine aminotransferase (ALT), aspartate aminotransferase (AST), alkaline phosphatase (ALP), gamma-glutamyl transferase (GGT), or total bilirubin levels exceeding 1.5 times the upper limit of the laboratory’s reference interval. Kidney function: Serum creatinine or cystatin C levels exceeding the laboratory’s reference interval. A known history of genetic disorders, malignancy, or autoimmune diseases. Obesity (body mass index ≥ 30 kg/m^2^). Any acute illness (e.g., infection, fever), surgery, blood transfusion, or hospitalization within the preceding four weeks. Chronic inflammatory or infectious diseases (e.g., hepatitis, tuberculosis). Based on these criteria, 35 individuals were excluded, resulting in a final cohort of 285 participants included in the analysis. The study protocol was approved by the Ethics Committee of the Zigong First People’s Hospital. All methods were performed in accordance with the relevant guidelines and regulations. Written informed consent was obtained from all participating adults.The flow chart of this study is shown in Figure 1.

### Flow cytometry analysis of nCD64 and mHLA-DR

The nCD64 index and mHLA-DR were measured using a Beckman Coulter Navios flow cytometer (Beckman Coulter, California, USA) and analyzed with Kaluza Analysis Software version 2.1. Following standard venipuncture protocol, approximately 2 mL of whole blood was collected from each participant into a K_2_ EDTA tube. Within 4 hours of collection, a 50 μL aliquot was stained with a pre-mixed antibody cocktail containing the following mouse anti-human monoclonal antibodies (all from Beckman Coulter unless specified): CD45-PC7 (clone J.33), CD14-APC750 (clone RM052), CD13-ECD (clone SJ1D1), CD64-PE (clone 22), and HLA-DR-FITC (clone Immu-357). The sample was incubated at room temperature in the dark for 30 minutes. Erythrocytes were then lysed using 2 mL of Optilyse C Lysing Solution (Beckman Coulter) according to the manufacturer’s instructions. After a wash step, cells were resuspended in 500 μL of phosphate-buffered saline for acquisition.

The flow cytometer was calibrated daily using Flow-Set Pro and Flow-Check Pro fluorospheres (Beckman Coulter). For multicolor compensation, single-stain controls were prepared using UltraComp eBeads (Thermo Fisher Scientific) stained with each antibody individually prior to sample acquisition.

**Fig. 2:**
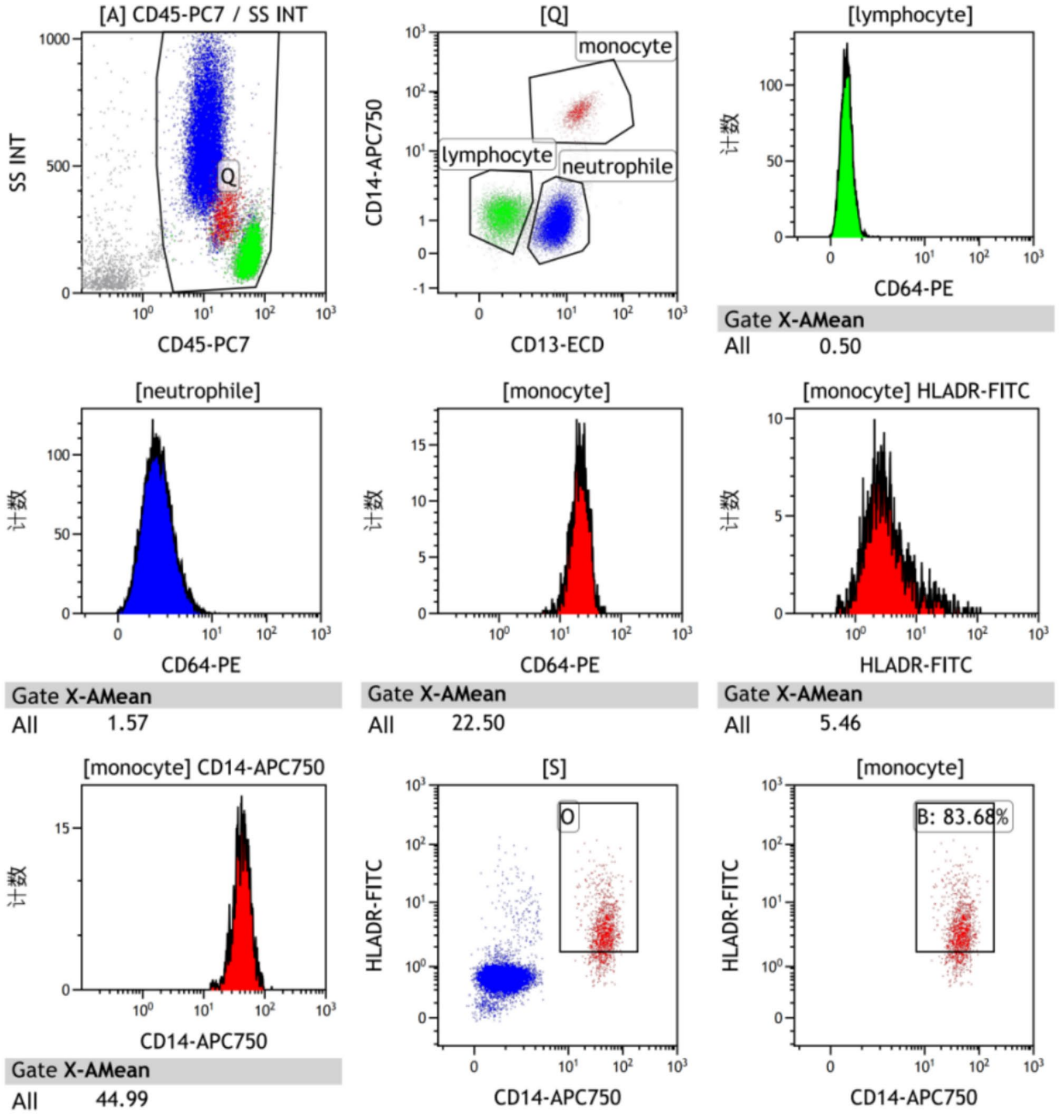
Schematic diagram of the flow cytometry analyses of nCD64 index and mHLA-DR.

Data from at least 15,000 nucleated cell events were acquired. The analysis was performed as follows (see Figure 2 for representative plots): Cells were first gated on a plot of Side Scatter (SSC) versus the pan-leukocyte marker CD45 to exclude debris and select the total population of nucleated leukocytes (Region A). Within the A gate, monocytes, neutrophils, and lymphocytes were identified and isolated using a combination of CD14 and CD13 expression (Region Q): Monocytes were defined as CD14+/CD13+. Neutrophils were defined as CD14-/CD13+. Lymphocytes were defined as CD14-/CD13-.

For CD64, the Median Fluorescence Intensity (MFI) was recorded within the pre-gated neutrophil, monocyte, and lymphocyte populations, yielding nCD64-MFI, mCD64-MFI, and LyCD64-MFI, respectively. The nCD64 index was then calculated using the formula: nCD64 index=[nCD64-MFI/Ly CD64-MFI]/[m CD64-MFI/nCD64-MFI]^7^.

For mHLA-DR, analysis was performed on the pre-gated monocyte population. The percentage of HLA-DR-positive monocytes was determined using a fluorescence-minus-one (FMO) control to set the positive threshold. The MFI of HLA-DR on the positive monocyte population was also recorded.

### Hematological analysis

Complete blood count (CBC) analysis was performed on all blood samples to screen participants and to derive inflammatory indices. Following venipuncture, the EDTA-anticoagulated whole blood samples were kept at room temperature and analyzed within 2 hours of collection to ensure cell stability and result accuracy. The analysis was conducted using a Sysmex XN-9000 automated hematology analyzer (Sysmex Corporation, Kobe, Japan) with the manufacturer-provided original reagents, including diluents, lysing agents, and calibrators. The instrument underwent daily quality control procedures using commercial quality control materials provided by the manufacturer to ensure precision and accuracy. The following parameters were recorded for each participant and used for subsequent calculations: white blood cell count (WBC), absolute neutrophil count (NEU), absolute lymphocyte count (LYM), absolute monocyte count (MON), platelet count (PLT), and hemoglobin (Hb). Derived inflammatory indices - including the neutrophil-to-lymphocyte ratio (NLR), platelet-to-lymphocyte ratio (PLR), systemic immune-inflammation index (SII), derived NLR (d-NLR), and lymphocyte-to-monocyte ratio (LMR) - were calculated from these primary CBC parameters.

### Establishing reference intervals

The procedure for determining reference intervals followed the recommendations outlined in the CLSI EP28-A3C guideline^8^. Initially, normality of distribution was assessed using skewness and kurtosis tests. Data deviating from normality were transformed via the Box–Cox method to approximate a normal distribution. Outliers were identified and removed using Tukey’s method, where values outside the range [P25-1.5×IQR, P75+1.5×IQR] were considered outliers. Participants were then stratified by sex and age. The standard normal deviation test (Z-test) was applied to evaluate whether reference intervals could be merged across subgroups. Finally, the 95% reference intervals were calculated using parametric methods for normally distributed data and nonparametric approaches for data that remained non-normal after transformation.

### Reference interval verification

The age-adjusted reference intervals were validated using 40 healthy adults from each age group. The obtained intervals were considered verified if fewer than 5% of individuals in the validation sets fell outside the proposed reference limits.

### Statistical analysis

All analyses were performed using Stata 15.0 (StataCorp, College Station, TX, USA) and SPSS Statistics for Windows version 22.0 (IBM Corp., Armonk, NY, USA). Continuous variables are presented as mean ± standard deviation (SD). Normality was evaluated with the Kolmogorov–Smirnov test. For non-normally distributed variables (nCD64 index and mHLA-DR), nonparametric tests were employed for comparisons across age groups. The Mann–Whitney U test and Z-test were used to assess between-group differences, with a two-tailed P-value < 0.05 considered statistically significant.

## Results

### Distribution of data and elimination of outliers

**Fig. 3:**
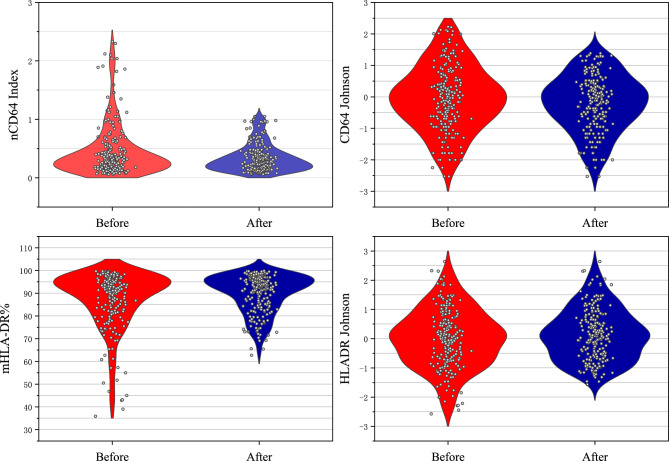
Violin plots for the data before and after outliers were eliminated by the Tukey method.

The initial distributions of the nCD64 index and mHLA-DR data were assessed for normality. The skewness and kurtosis values were 2.20 and 4.97 for the nCD64 index, and −1.79 and 3.39 for mHLA-DR, respectively, indicating significant deviation from a normal distribution based on the Skewness-Kurtosis test. A Box-Cox transformation was subsequently applied to normalize the datasets. Following transformation, the respective P-values increased to 0.737 for the nCD64 index and 0.955 for mHLA-DR, and the skewness/kurtosis values approached zero (−0.01 & −0.37 for nCD64; −0.01 & −0.16 for mHLA-DR), confirming an approximate normal distribution. A summary of the parameters before and after transformation is provided in Table 1. Outliers were then identified and removed using Tukey’s method. Detailed parameters before and after this elimination step are presented in Figure 3 and Table 1.

**Table 1:** The data before and after outliers were eliminated by the Tukey method.

Parameter	Before eliminate						After eliminate					
	N	P_25_	P_75_	IQR	Max	Min	N	P_25_	P_75_	IQR	Max	Min
nCD64	285	0.18	0.54	0.36	2.33	0.06	269	0.17	0.44	0.27	1.05	0.06
mHLA-DR	285	89.04	97.43	8.39	99.85	35.86	265	90.43	97.55	7.12	99.85	78.53

### Establishment of the reference interval

Participants were stratified by sex (male, female) and age, with a biologically relevant cut-off set at 50 years to reflect the onset of more pronounced immunosenescence^9^. The potential influence of these factors as grouping variables was evaluated. Analysis of variance (ANOVA) indicated that neither sex nor age were significant dividing factors for mHLA-DR levels (p > 0.05). For the nCD64 index, however, a significant difference was observed among age groups (p < 0.05). Subsequent application of the standard normal deviate test (Z-test) confirmed the necessity to establish age-specific reference intervals for the nCD64 index, as detailed in Table 2. The 95% reference intervals (RIs) for both biomarkers, after outlier exclusion, were calculated using a nonparametric method for the overall study population (aged 20–90 years). All established RIs are presented in Table 3.

**Table 2:** The nCD64 index results of pairwise comparison of age subclass by standard normal deviate test.

Age groups (year)		F value	P value	*Z*	Z*
20–50(n=124)	51–90(n=145)	26.881	<0.001	5.225	4.775
$$Z=\frac{|\overline{\mathrm{X} }1-\overline{\mathrm{X} }2|}{{\left[\left(\frac{{S1}^{2}}{N1}\right)-\left(\frac{{S2}^{2}}{N2}\right)\right]}^\frac{1}{2}},$$$${Z}^{*}=3{\left[\left(N1+N2\right)/240\right]}^\frac{1}{2}.$$ (*N*1≥120, *N*2≥120) $$\overline{\mathrm{X} }1$$ and $$\overline{\mathrm{X} }2$$ are the observed means of the two subgroups, S1 and S2 are the observed variances, and *N*1 and *N*2 are the number of reference intervals in each subclass, respectively. If the calculated Z exceeds Z*, they recommend partitioning.

**Table 3:** The nCD64 index and mHLA-DR reference interval calculate by nonparametric methods.

Parameter	nCD64 index (95% RI: P0-P95)		mHLA-DR(%)(95% RI: P5-P100)
Age (range, year)	20–50	51–90	20–90
Nonparametric method RI	0.00–0.59.00.59	0.00–0.97.00.97	82.8–100.0.8.0
RI (90%CI)	0.00–0.59.00.59 (0.45–0.69.45.69)	0.00–0.97.00.97 (0.81–0.99.81.99)	82.8–100.0.8.0 (81.2–84.7.2.7)

RI: reference interval defined by nonparametric method (P95).

### Verification of reference intervals

The validity of the proposed reference intervals was tested using an independent verification cohort. This cohort comprised 80 additional healthy individuals (aged 20–90 years) from the same local population, who were screened with the same exclusion criteria detailed in Section "Patients." and were not part of the main reference population. They were equally divided into a younger (20–50 years, n=40) and an older (51–90 years, n=40) subgroup. The nCD64 index and mHLA-DR were measured for all individuals. As shown in Table 4, over 95% of the results from this verification cohort fell within the corresponding established reference intervals for all subgroups, thereby successfully validating the proposed RIs.

**Table 4:** The comparison results of verify population by different nCD64 index and mHLA-DR reference intervals.

	nCD64 index		mHLA-DR(%)
Age (range, year)	20–50 (n = 40)	51–90 (n = 40)	20–90 (n = 80)
	RI (0.00–0.59.00.59)	RI (0.00–0.97.00.97)	RI (82.8–100.0.8.0)
Negative%	96.3	95.3	95.9
Positive%	3.7	4.7	4.1

### Correlation analysis between age and nCD64 index

**Fig. 4:**
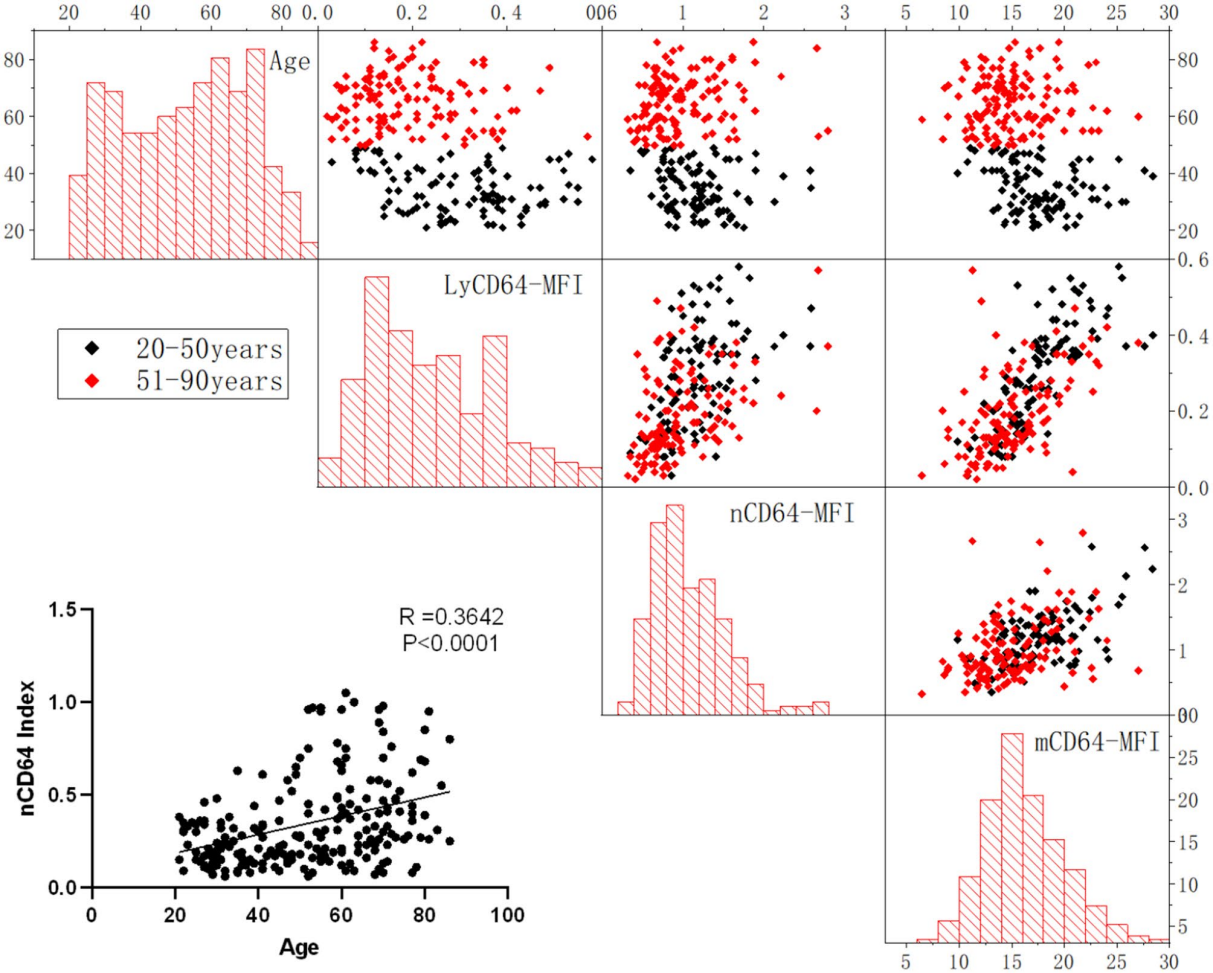
Scatter diagram for interrelationships between age, LyCD64-MFI, nCD64-MFI, mCD64-MFI and nCD64 index.

**Fig. 5:**
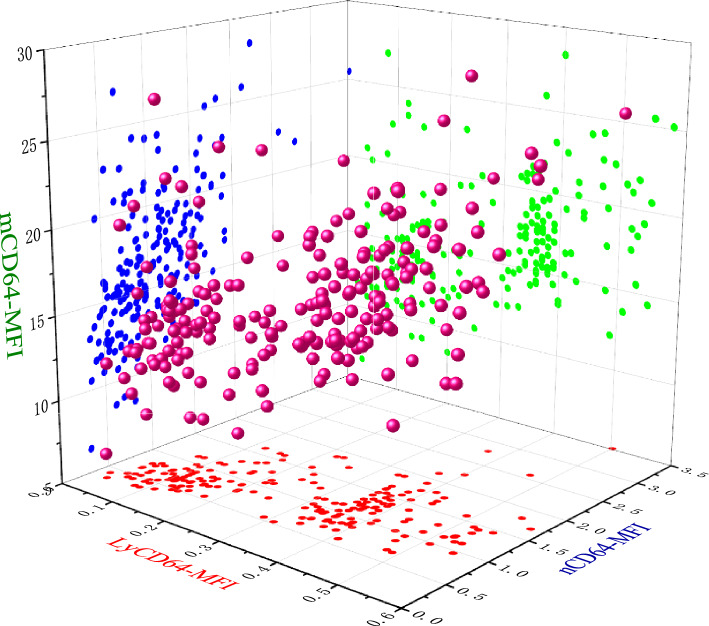
3D scatter diagram for interrelationships between LyCD64-MFI, nCD64-MFI and mCD64-MFI.

In order to study whether there is a trend change of nCD64 index with the increase of age, we analyzed the correlation analysis between age and nCD64 index, CD64 MFI on lymphocyte, neutrophil and monocyte, we found that mCD64-MFI had a negative correlation with age (r = 0.3365, P < 0.00.01), and the others (nCD64-MFI and LyCD64-MFI) also have negative correlation with age(r = 0.2109, P = 0.0014; r = 0.3736, P < 0.0001, respectively). nCD64 index (CD64 MFI calculated value) have positive correlation with age (r = 0.3642, P < 0.0001), their interrelationships are shown in Figure 4. CD64 MFI interacted on lymphocytes, neutrophils and monocytes, and all of them increased with the increase of others. CD64 MFI on lymphocytes was positively correlated with CD64 MFI on neutrophils and on monocyte (r = 0.5585, P < 0.0001; r = 0.462, P < 0.0001, respectively), CD64 MFI on neutrophils was also positively correlated with CD64 MFI on monocyte (r = 0.4715, P < 0.0001), their interrelationships are shown in 3D graph (Fig 5).

### Correlation analysis between blood routine indexes and nCD64 index, mHLA-DR%

We further studied the correlation of blood routine indexes and derivative indexes (including PLR, SII, NLR, d-NLR, LMR) with nCD64 index and mHLA-DR%, we calculate the derivative indexes according to the following formula: PLR = number of platelet (PLT)/Absolute number of lymphocytes (LYM-abs), SII = number of PLT × Absolute number of neutrophils (NEU-abs)/LYM-abs, NLR = NEU-abs/LYM-abs, d-NLR = [White blood cells (WBC) - NEU-abs]/LYM-abs, LMR = LYM-abs/Absolute number of monocytes (Mono-abs). we found that no correlation between nCD64 index and mHLA-DR% and all the indicators of blood routine (P>0.05).

### Comparison with published reference intervals

To contextualize the reference intervals established in the present study, a comparison with published data from other regions in China was conducted. The key findings from other studies regarding the nCD64 index and mHLA-DR% in healthy adults are summarized in Table 5 and Table 6, respectively.

**Table 5:** Reference intervals of nCD64 in adults: comparison among different regions in China.

ID	Author	Year	region	n	Age(years)	Reagent manufacturer and instrument type	nCD64 index (M±S) or M(P25-P75)	nCD64 index (RI)
1	Yanting Gao^10^	2022	Shanghai	36	32–95	Beckman coulter; Cytek Northern lights-CLC	0.33±0.16	0–0.59.59
2	Jianhua Yu^11^	2021	Guangdong	40	17–85	Beckman coulter; beckman coulter navios	0.33(0.16–0.54.16.54)	0–0.74.74
3	YuXi Shang^12^	2022	Beijing	26	22–76	Trillium diagnostics; BD FACS cantoII	0.71±0.31	0–1.21.21
4	Biao Wang^13^	2022	Hubei	40	35–60	Becton dickinson; BD FACS calibur	0.71±0.21	0–1.05.05

**Table 6:** Reference intervals of mHLA-DR in adults: comparison among different regions in China.

ID	Author	Year	region	n	Age(years)	Reagent manufacturer and instrument type	mHLADR% (M±SD) or (P25-P75)	mHLADR (RI)
1	Jianhua Yu^11^	2021	Guangdong	40	17–85	Beckman coulter; Beckman coulter navios	91.2(84.63–95.75.63.75)	78.0–100.0
2	Tianrong Zhou^14^	2021	Yunnan	20	47–64	Becton dickinson; BD FACSCalibur	96.07(93.2–98.8.2.8)	90.4–100.4
3	Shujun Zhou^15^	2013	Jiangsu	38	40–88	Becton dickinson; BD FACSCalibur	69.6±8.7	55.5–100.5

For the nCD64 index, data from four independent studies were compared (Table 5). The reported upper limits of the reference intervals (RI) showed variation, ranging from 0.59 to 1.21. The mean or median values also differed, spanning from 0.33 to 0.71.

For mHLA-DR%, data from three studies were analyzed (Table 6). The reported reference intervals for this biomarker also exhibited variability. The lower limits of the RIs ranged from 55.5% to 90.4%, while the upper limits were consistently reported as 100% across studies. The central tendency measures (mean/median) varied from 69.6% to 96.07%.

## Discussion

CD64 is primarily expressed on the antigen-presenting cells. Under normal condition, neutrophil CD64 expression is low but increases rapidly within 4–6 hours after infection, serving as an early indicator of bacterial infection unaffected by hormones or antibiotics. CD64 detection can be performed using three methods: the percentage of CD64 positive cell, CD64 average fluorescence intensity and CD64 index. Among these, CD64 index minimizes the influence of external factors such as research objects and measuring instruments, offering higher sensitivity and specificity^16^. Therefore, this study used the nCD64 index to establish a reference interval for adults. The results showed that the mean value of nCD64 index was 0.57 in healthy individuals aged 20–50 years old, and 1.0 in those aged 51–90 years old. The nCD64 index gradually increased with age and showed a positive correlation with age (r = 0.3642, P < 0.0001). Many studies have indicated that the aging process is often accompanied by a state of chronic low-grade inflammation, which is described as inflammatory aging^17,18^. Unlike acute inflammation or infection, chronic low-grade inflammation is characterized by a long-term mild elevation of the representative inflammatory factors such as C-reactive protein and interleukin 6. Inflammatory aging may result from the accumulation of endogenous macromolecules or cellular debris, an increase in senescent cells and aging-related secretory phenotypes, declining immune function, alterations in microorganisms and their metabolites, and enhanced activity of the coagulation system^10,19^. Since nCD64 index is itself an indicator of inflammation, the findings of this study are consistent with the concept of inflammatory aging. However, there was no correlation between mHLA-DR and age in this study, this finding is consistent with evidence suggesting that the expression of HLA-DR on classical monocyte subsets remains stable across different age groups in healthy adults^20^. This indicates that despite the presence of age-related, chronic low-grade inflammation, it may have a minimal impact on this specific aspect of monocyte antigen-presenting function. Specifically, our data suggest there is neither a progressive immunosuppression nor a heightened immune activity in monocytes with advancing age within a healthy population.

In this study, it was observed that the CD64 MFI on lymphocytes, neutrophils and monocytes exhibited a slight gradual increase with age. Further analysis showed that the CD64MFI values among these cell types interact, with the value in one cell type increasing in correlation with the fluorescence intensity of the other two. These findings indicate that CD64 MFI can be influenced by variables such as sample type, instrument voltage, and environmental conditions. Consequently, the observed trend of increasing CD64 MFI on lymphocytes and granulocytes with age is likely artifactual. Therefore, in order to minimize the impact of these external confounding factors, the CD64 index is currently recognized as the best method.

To evaluate the broader applicability of the reference intervals established in this study, we compared our findings with published data from other regions in China. As summarized in the Results section (Tables 5 and 6), there exists considerable variability in the reported reference intervals for both the nCD64 index and mHLA-DR% among healthy adult populations across studies. This observed heterogeneity is not entirely unexpected and can be attributed primarily to methodological differences. For the nCD64 index, the variation appears to correlate with the analytical platform; studies employing instruments and reagents from different manufacturers report distinct reference limits. More pronounced variability was noted for mHLA-DR%. The measurement of mHLA-DR as a positive percentage is inherently sensitive to a confluence of technical factors. These include the specific characteristics of the fluorochrome-antibody conjugate, instrument settings, and the applied gating strategy^21^. Spectral overlap during multicolor staining further contributes to this variability, explaining why studies using different instrumentation protocols may yield divergent reference ranges^21^.

Therefore, the central implication of our comparative analysis is that reference intervals for mHLA-DR% and, to a lesser extent, the nCD64 index, are highly method-dependent. Our findings reinforce the critical need for clinical laboratories to establish and validate their own reference intervals based on their specific instrumentation, reagents, and standard operating procedures. The intervals presented here are robust for our local population and methodological context. For broader application, method-specific verification is strongly recommended to ensure accurate clinical interpretation across different testing platforms.

The peripheral blood inflammatory indicators, such as systemic immune inflammatory index (SII), neutrophil to lymphocyte ratio (NLR), derived neutrophil to lymphocyte ratio (d-NLR), platelet to lymphocyte ratio (PLR), and lymphocyte to monocyte ratio (LMR), have been used as useful diagnostic or prognostic markers for various inflammatory diseases^22–24^. Accordingly, this study performed correlation analysis between these indicators and both the nCD64 index and mHLDR%. The results revealed no significant correlations between the inflammatory markers and either nCD64 index or mHLDR%, indicating that nCD64 index and mHLDR% may serve as independent risk indicators in inflammatory diseases.

The reference intervals established in this study are based on a healthy adult population from a specific region in China. It is important to note that biomarker levels, including immune markers like nCD64 and mHLA-DR, may be influenced by ethnic, genetic, and environmental factors. For instance, a systematic review highlights that immune responses exhibit considerable inter-individual variation influenced by a complex interplay of factors, including genetics, age, sex, and environmental exposures, which underscores the necessity of establishing population-specific reference values^25^. Therefore, the intervals reported here should be validated or adjusted when applied to populations with distinct ethnic backgrounds or from different geographic regions. Future multi-center studies involving diverse populations are warranted to establish more universally applicable reference intervals or to define appropriate correction factors.

## Conclusion

The reference intervals of nCD64 index and mHLDR% established in this study are expected to support improved clinical evaluation and treatment of adult patients in China, A better understanding of age-related phenotypic changes is essential for the accurate identification of infectious diseases, particularly as these markers serve as critical independent risk indicators. Furthermore, future studies should prioritize extending this research to children under 20 years of age.

## Data Availability

The data supporting the findings of this study are available from the corresponding author upon reasonable request.
